# HIV Services Provided by STD Programs in State and Local Health Departments — United States, 2013–2014

**DOI:** 10.15585/mmwr.mm6613a2

**Published:** 2017-04-07

**Authors:** Kendra M. Cuffe, Precious Esie, Jami S. Leichliter, Thomas L. Gift

**Affiliations:** ^1^Division of STD Prevention, National Center for HIV/AIDS, Viral Hepatitis, STD, and TB Prevention, CDC; ^2^Oak Ridge Institute for Science and Education.

The incidence of human immunodeficiency virus (HIV) infection in the United States is higher among persons with other sexually transmitted diseases (STDs), and the incidence of other STDs is increased among persons with HIV infection (*1*). Because infection with an STD increases the risk for HIV acquisition and transmission (*1*–*4*), successfully treating STDs might help reduce the spread of HIV among persons at high risk (*1*–*4*). Because health department STD programs provide services to populations who are at risk for HIV, ensuring service integration and coordination could potentially reduce the incidence of STDs and HIV. Program integration refers to the combining of STD and HIV prevention programs through structural, service, or policy-related changes such as combining funding streams, performing STD and HIV case matching, or integrating staff members (*5*). Some STD programs in U.S. health departments are partially or fully integrated with an HIV program (STD/HIV program), whereas other STD programs are completely separate. To assess the extent of provision of HIV services by state and local health department STD programs, CDC analyzed data from a sample of 311 local health departments and 56 state and directly funded city health departments derived from a national survey of STD programs. CDC found variation in the provision of HIV services by STD programs at the state and local levels. Overall, 73.1% of state health departments and 16.1% of local health departments matched STD case report data with HIV data to analyze possible syndemics (co-occurring epidemics that exacerbate the negative health effects of any of the diseases) and overlaps. Similarly, 94.1% of state health departments and 46.7% of local health departments performed site visits to HIV care providers to provide STD information or public health updates. One fourth of state health departments and 39.4% of local health departments provided HIV testing in nonclinical settings (field testing) for STD contacts, and all of these programs linked HIV cases to care. STD programs are providing some HIV services; however, delivery of certain specific services could be improved.

Given the likely synergistic relationship between STDs and HIV and the potential role of STDs in HIV acquisition (*2*–*4*), public STD programs, including clinics and health department programs that provide STD services, could be an important venue for providing HIV services to populations at high risk (*6*) and persons not well connected to health care. One study, a convenience sample of 10 U.S. jurisdictions, found that public STD clinics diagnosed approximately 10%–35% of HIV cases within those jurisdictions (*6*). The extent of the provision of other HIV services by public STD programs has not been assessed at a national level. This national-level report examines the current state of HIV services provided by public STD programs.

This report included two separate types of respondents: local health departments and state health departments. First, a sample of 311 local health departments was drawn from the 1,225 local health departments that indicated that they provided STD screening or treatment in a 2010 National Profile of Local Health Departments survey. This sample included cities and counties with the 50 highest number of reported cases or rates of STDs in 2010 and the six cities directly funded by CDC’s Division of STD Prevention.* Second, all 50 states are directly funded by CDC’s Division of STD Prevention and were included in the state sample. From December 2013 to January 2014, a survey was sent to primary contacts of the sampled STD programs in 1) local health departments, including health departments within U.S. cities, counties, and other sub-state regions (i.e., county clusters) and 2) state health departments across the United States. Weights based on U.S. Census region, jurisdiction size, and nonresponse were used in all analyses focusing on local health departments. Jurisdiction population size was categorized as small (<50,000), medium (50,000–499,999) and large (≥500,000). The extent of HIV field testing, linkage to care, and follow-up for persons testing positive for HIV during partner services, program visits to HIV care providers, and epidemiology and surveillance activities related to HIV were assessed. Provision of HIV services by health department type (state versus local) was examined using logistic regression models; among local health departments, chi-square analyses were used to determine whether jurisdiction size was associated with type of HIV service provided. 

The response rate was 47.6% for local health departments and 60.7% for state health departments. The largest proportion of responding local health departments were from the South (35.8%) followed by the Midwest (28.4%), West (20.9%), and Northeast (14.9%) U.S. Census regions, whereas the largest proportion of responding state health departments were from the West (30.3%) followed by the Northeast (27.3%), Midwest (21.2%), and South (21.2%) regions. Among local health departments, 39.2% had jurisdictions classified as small, 35.1% as medium, and 25.7% as large.

Differences were identified among HIV services provided by STD programs in local and state health departments (Table 1). A significantly higher percentage of state health departments conducted visits to HIV care providers (94.1%) than did local health departments (46.7%). A higher proportion of surveyed state health departments reported targeting prevention activities to populations at high risk (92.3%) than did local health departments (58.4%). A higher percentage of state health departments than local health departments matched STD case report data with HIV data to analyze syndemics and overlaps (73.1% versus 16.1%). Nonsignificant differences were also found between local and state health departments for some HIV services. For example, 25.0% of local health departments and 39.4% of state health departments provided HIV field testing for STD contacts. All state and local health departments that field-tested STD contacts for HIV linked to care any persons with HIV identified during partner services field-testing. A majority of local health departments reported that disease intervention specialists or communicable disease investigators were responsible for performing linkages to care (52.3%), followed by public health nurses (31.9%) and persons in other job categories (15.8%). In state health departments, disease intervention specialists or communicable disease investigators performed most of the linkages to care (83.3%), followed by public health nurses (8.3%) and community health outreach workers (8.3%).

**TABLE 1:** HIV services provided by STD programs in local and state health departments* — United States, 2013–2014

Services	Local health departments (unweighted n = 148)		State health departments (unweighted n = 33)		p value
	No.	Weighted % (95% CI)	No.	% (95% CI)	(Local versus state)
**HIV field testing for STD contacts (n = 1,225)**					0.11
Yes	35	25.0 (17.7–34.1)	13	39.4 (23.9–57.4)	
No	113	75.0 (65.9–82.3)	20	60.6 (42.6–76.1)	
**Linkage to care for persons found to be HIV+ during partner-services field testing (n = 306)**					0.07
Yes, by health department staff members (n = 200)	23	65.4 (45.8–80.9)	12	92.3 (58.9–99.0)	0.05
DIS/CDI	13	52.3 (29.8–73.9)	10	83.3 (50.7–96.1)	
Public health nurse	7	31.9 (14.8–55.8)	1	8.3 (1.1–43.4)	
Community health outreach worker	0	—	1	8.3 (1.1–43.4)	
Other	3	15.8 (4.4–43.3)	0	—	
Yes, by referral	12	34.6 (19.1–54.2)	1	7.7 (1.0–41.1)	
No	0	—	0	—	
**Follow-up of HIV patients who have been linked to care (n = 306)**					0.86
Yes	32	89.3 (70.6–96.7)	12	92.3 (58.9–99.0)	
No	2	5.9 (1.4–21.4)	1	7.7 (1.0–41.1)	
Don't know	1	4.8 (0.7–27.5)	0	—	
**Program visited HIV care providers (n = 516)**					<0.01
Yes	38	46.7 (33.2–60.8)	16	94.1 (66.1–99.2)	
No	28	53.3 (39.2–66.8)	1	5.9 (0.8–33.9)	
**Match STD case report data with HIV data to analyze syndemics/overlaps (n = 947)**					<0.01
Yes	19	16.1 (9.7–25.5)	19	73.1 (52.4–87.0)	
No	95	83.9 (74.5–90.3)	7	26.9 (13.0–47.6)	
**Target prevention activities to population at high risk (n = 947)**					0.01
Yes	71	58.4 (47.6–68.4)	24	92.3 (72.7–98.2)	
No	43	41.6 (31.6–52.4)	2	7.7 (1.8–27.3)	
**Publish and disseminate data on a health department website at least annually (n = 947)**					<0.01
Yes	30	22.2 (14.8–31.9)	18	69.2 (48.6–84.3)	
No	84	77.8 (68.1–85.2)	8	30.8 (15.7–51.4)	

**Abbreviations:** CDI = communicable disease investigator; CI = confidence intervals; DIS = disease intervention specialist; HIV = human immunodeficiency virus; HIV+ = HIV positive; STD = sexually transmitted diseases.

* Table shows unweighted numbers, weighted column percentages, and weighted 95% CIs for local health departments. Weighted numbers are included next to each variable for local health departments. Unweighted numbers, column percentages, and 95% CIs are shown for state health departments.

For local health departments only, the delivery of HIV services by STD programs was examined by jurisdiction size (Table 2). Local health departments with small jurisdictions were significantly less likely to offer HIV field testing for STD contacts (11.4%) than were those with medium (37.8%) and large (39.0%) jurisdictions. Performing site visits to HIV care providers was significantly associated with jurisdiction size and was more commonly reported by large (95.6%) and medium (59.0%) than small jurisdictions (26.0%).

**TABLE 2:** HIV services provided by STD programs in local health departments,* by jurisdiction size — United States, 2013–2014

Services	Unweighted no.	Jurisdiction size (population)			p value
		<50,000	50,000–499,999	≥500,000	
		Weighted % (95% CI)	Weighted % (95% CI)	Weighted % (95% CI)	
**HIV field testing for STD contacts (n = 1,225)**					<0.01
Yes,	35	11.4 (2.5–20.2)	37.8 (23.0–52.4)	39.0 (21.4–56.6)	
No	113	88.6 (79.8–97.5)	62.2 (47.6–76.9)	61.0 (43.4–78.6)	
**Linkage to care for persons found to be HIV+ during partner-services field testing (n = 306)**					0.52^†^
Yes, by health department staff members (n = 200)	23	51.7 (8.6–94.8)	72.4 (48.2–96.5)	55.0 (23.7–86.2)	
DIS/CDI	13	68.9 (13.1–100.0)	43.7 (12.2–75.1)	80.3 (52.2–100.0)	
Public health nurse	7	31.1 (0.0–86.9)	35.7 (6.5–64.9)	9.4 (0.0–29.0)	
Community health outreach worker	0	—	—	—	
Other	3	0.0	20.6 (0.0–47.7)	10.3 (0.0–31.5)	
Yes, by referral	12	48.3 (5.2–91.4)	27.6 (3.5–51.8)	45.0 (13.8–76.2)	
No	0	—	—	—	
**Follow-up of HIV patients who have been linked to care (n = 306)**					<0.001
Yes	32	100.0 (0.0–100.0)	83.3 (64.4–100.0)	100.0 (0.0–100.0)	
No	2	—	9.2 (0.0–22.3)	—	
Don’t know	1	8.7 (0.2–17.3)	5.0 (0.0–12.2)	5.7 (0.0–13.5)	
**Program visited HIV care providers (n = 516)**					<0.01
Yes	38	26.0 (6.5–45.5)	59.0 (35.2–82.8)	95.6 (86.8–100.0)	
No	28	74.0 (54.5–93.5)	41.0 (17.2–64.8)	4.4 (0.0–13.2)	
**Match STD case report data with HIV data to analyze syndemics/overlaps (n = 947)**					0.23
Yes	19	18.8 (5.9–31.7)	10.4 (0.0–20.8)	30.7 (11.3–50.1)	
No	95	81.2 (68.3–94.1)	89.6 (79.2–100.0)	69.3 (49.9–88.7)	
**Target prevention activities to population at high risk (n = 947)**					0.36
Yes	71	55.0 (38.9–71.1)	58.1 (41.8–74.3)	78.8 (61.8–95.9)	
No	43	45.0 (28.9–61.1)	41.9 (25.7–58.2)	21.2 (4.1–38.2)	
**Publish and disseminate data on a health department website at least annually (n = 947)**					<0.01
Yes	30	8.0 (0.24–15.8)	34.1 (18.4–49.7)	37.8 (17.9–57.7)	
No	84	92.0 (84.2–99.8)	65.9 (50.3–81.6)	62.2 (42.3–82.1)	

**Abbreviations:** CDI = communicable disease investigator; CI = confidence intervals; DIS = disease intervention specialist; HIV = human immunodeficiency virus; HIV+ = HIV positive; STD = sexually transmitted diseases.

* Table shows unweighted numbers, weighted column percentages, and weighted 95% CIs for local health departments. Weighted numbers are included next to each variable for local health departments.

^†^ p value represents differences detected between DIS/CDI and public health nurses only given the zero values in the community health outreach worker and other categories.

## Discussion

Many public STD programs in state and local health departments reported that they provided multiple HIV services. Among programs that provided HIV field testing services, all provided linkages to HIV care, with most using health department staff members to provide this linkage, rather than simply providing a referral. Also, approximately two thirds of STD programs in state and local health departments provided follow-up for newly identified HIV cases that were linked to care. Finally, the majority of state health departments reported that they visited HIV care providers, matched STD case report data with HIV data to analyze syndemics and/or overlaps, targeted prevention activities to populations at high risk, and published and disseminated surveillance data on the health department at least annually.

Despite encouraging progress, areas where improvements in provision of HIV services by STD programs might be beneficial were also identified. For example, fewer than half of STD programs indicated that they provided HIV field testing for STD contacts; in particular, local health departments in small jurisdictions were unlikely to provide HIV field testing. HIV field testing is especially important for syphilis cases, because the sexual networks of syphilis patients overlap with those of persons with HIV infection, and approximately half of men with syphilis have concomitant HIV infection (*7*). Furthermore, fewer than one in six STD programs in local health departments matched STD and HIV case data, and fewer than one in four disseminated surveillance data on a health department website. Finally, fewer local health departments targeted prevention to those at high risk than did state health departments.

The findings in this report are subject to at least four limitations. First, the response rate for local health departments was only 47.6%; however, weights were applied to local health department data to adjust for nonresponse. Second, the survey did not assess whether STD and HIV programs were integrated, and if so, the extent of integration. Third, these survey items were limited to questions about STD program activities, and it is possible that other government or community-based organizations are providing such services. A more comprehensive assessment of all HIV activities might yield a better picture of what services are being provided in a community. Finally, the survey did not ask about HIV clinical services, such as HIV testing in STD clinics.

Given the recognized association between STDs and HIV risk, STD programs can and do play a role in HIV prevention (*1*–*4*). These findings highlight some of these important efforts as well as suggest areas for possible expansion. It is important to note that health department public clinics that provide STD services, particularly STD clinics, serve populations at risk for HIV and often serve as surveillance sites for both STDs and HIV (*8*). Such clinics might serve as access points to deliver HIV prevention services for persons at risk who might otherwise lack access to health care. Also, the importance of public STD clinics to support HIV surveillance through reporting new HIV cases identified during partner services and to provide HIV services to STD cases and their partners was demonstrated early on in the HIV epidemic during the late 1980s (*9*). Therefore, data collected through STD programs often illuminate important opportunities for enhancing STD and HIV surveillance data and helping inform future decisions affecting STD and HIV prevention programs. Finally, numerous STD clinics have been closed for reasons that include budget decreases (*10*); these closures might impact HIV services, increasing the importance of health department visits to HIV care providers (e.g., to remind providers of STD testing recommendations in jurisdictions lacking STD clinics) and STD/HIV case data matching (*10*). Evaluating the impact of STD program reduction on these services can help identify the impact of STD program reduction on HIV prevention and linkage to care.

**Summary**

**What is already known about this topic?**

STD programs in health departments often provide both STD services and HIV-specific services, including partner services such as interviewing and testing sex partners.

**What is added by this report?**

Findings from this report show that STD programs often provide integral HIV specific services including HIV field testing for STD contacts and linking those found to be HIV-positive to care. This report also illustrates that state health departments often perform visits to HIV providers to provide STD information or public health updates as well as perform epidemiologic activities, including matching STD cases report data with HIV data to analyze interactions between the diseases or overlaps.

**What are the implications for public health practices?**

STD programs can play an essential role in reducing HIV transmission among patients with STD diagnoses. Front line interaction with STD patients and contacts at high risk for HIV provides opportunities for quicker HIV testing and linkage to care. STD programs might also gain important insights in STD and HIV epidemiology as well as possible interactions between the diseases by matching and analyzing STD case report and HIV data, to the extent such data sharing is possible.

## Footnotes

* San Francisco, California; Los Angeles, California; Chicago, Illinois; Baltimore, Maryland; Philadelphia, Pennsylvania; and New York City, New York.
